# Direct Infection and Replication of Naturally Occurring Hepatitis C Virus Genotypes 1, 2, 3 and 4 in Normal Human Hepatocyte Cultures

**DOI:** 10.1371/journal.pone.0002660

**Published:** 2008-07-16

**Authors:** Martina Buck

**Affiliations:** 1 Department of Medicine and Moores Cancer Center, University of California, La Jolla, California, United States of America; 2 Department of Medicine, VA Healthcare Center, San Diego, California, United States of America; Institut Pasteur, France

## Abstract

**Background:**

Hepatitis C virus (HCV) infection afflicts about 170 million individuals worldwide. However, the HCV life cycle is only partially understood because it has not been possible to infect normal human hepatocytes in culture. The current Huh-7 systems use cloned, synthetic HCV RNA expressed in hepatocellular carcinoma cells to produce virions, but these cells cannot be infected with naturally occurring HCV obtained from infected patients.

**Methodology/Principal Findings:**

Here, we describe a human hepatocyte culture permissible to the direct infection with naturally occurring HCV genotypes 1, 2, 3 and 4 in the blood of HCV-infected patients. The culture system mimics the biology and kinetics of HCV infection in humans, and produces infectious virions that can infect naïve human hepatocytes.

**Conclusions/Significance:**

This culture system should complement the existing systems, and may facilitate the understanding of the HCV life cycle, its effects in the natural host cell, the hepatocyte, as well as the development of novel therapeutics and vaccines.

## Introduction

An estimated 170 million individuals have chronic hepatitis C virus (HCV) infection worldwide [1]. About 70% of infected individuals develop a chronic infection; for some, this includes fibrosis, cirrhosis, and hepatocellular carcinoma [2], [3]. Approximately, 10,000 deaths due to cirrhosis and several thousand more deaths due to hepatocellular carcinoma are attributed to HCV infection in the United States each year [4]. Unfortunately, there is no vaccine available and the current treatment for HCV infection, PEG-interferon-α in combination with ribavirin, achieves sustained responses only in ∼50% of treated patients [5].

The mechanisms responsible for the HCV life cycle in the liver of infected individuals are only partially understood because it has not been possible to infect normal human hepatocytes in culture with naturally occurring HCV obtained from HCV-infected patients [6], and because HCV is known to infect only humans and chimpanzees [4].

Recently, Lindenbach, Zhong , Wakita , Yi , Murakami and their coworkers [7]–[11], were able to replicate synthetic HCV RNA in hepatocellular carcinoma Huh-7-derived cells with the efficient production of HCV virions that were infectious to cultured Huh-7-derived cells [7]–[11] chimpanzees [9], [12] , and mice containing human liver grafts [12]. Importantly, virus recovered from these animals was highly infectious in cell culture [12]. Also, Aly and coworkers have developed a human hepatocyte cell line immortalized with human papilloma virus (HPV) 18/E6E7 susceptible to HCV infection [13]. The cell line's susceptibility to HCV infection was further increased by inhibiting the interferon regulatory factor-7 (IRF-7) [13].

The current Huh-7-derived HCV virions system uses non-naturally occurring, cloned HCV genotype 2a strain (JFH-1) [7], cloned HCV genotype 1a ( H77-S) containing five adaptive mutations [10] , or cloned HCV genotype 1b [11]. Limitations of this method are the use of cloned HCV, and the failure to infect these cells with naturally occurring HCV obtained from infected patients [14]

Further, hepatocellular carcinoma cell lines depart from normal human hepatocytes since they have abnormal proliferation, deregulated gene expression, dysfunctional mitochondria, and aberrant signaling and endocytosis pathways [15]–[20]. Relevant abnormalities of the Huh-7-derived cell lines include an absence of caveolin-1 and caveolin-2 [17], a mutated p53 (Y220C) [21], overexpression of the pituitary tumor transforming gene (PTTG) [22], cell cycle-independent expression of human telomerase reverse transcriptase (hTERT) [23], higher expression of glucose metabolism enzymes (glucose-6-phosphate 1-dehydrogenase and isocitrate dehydrogenase) and of a mitochondrial protein (dicarboxylate carrier) [24] . In addition, Huh-7 cells expressed the highest level of α-fetoprotein, a marker for hepatocellular carcinoma and de-differentiation, among 25 hepatocellular carcinoma cell lines tested [24]. Although the HPV-18/E6E7 immortalized human hepatocytes can be infected with serum-derived HCV, albeit at lower levels, it also over expresses hTERT [13], and like Huh-7-derived cells, its proliferation behavior should be that of tumor cells [24]. Consequently, any perturbation of these normal hepatocyte functions by the HCV infection cannot be studied completely and/or accurately in the Huh-7-derived HCV virions system or the HPV-18/E6E7 immortalized human hepatocytes [14].

Valuable studies by Fournier, Molina and their coworkers have allowed the culture of HCV in primary human hepatocytes [25]–[27]. However, this culture hepatocyte system had limited efficiency since less than 15% of the sera were infectious [25], the amplification was less than 1 log_10_ [25], [26], the infection declined after 8 days , it was detectable only in the cell layers until day-14, and there was no evidence of the production of infectious virions [25]–[27] Thus, there are still no effective means for directly culturing and significantly amplifying HCV from typical clinical specimens using differentiated normal human hepatocytes [4]. Here, we report the development of a normal human hepatocyte culture system permissible to the infection with, and physiologically significant amplification of, naturally occurring HCV.

## Results

### Infection of Primary Human Hepatocyte Cultures with Naturally Occurring HCV

We used primary human hepatocytes that were isolated from normal liver explants, and sera from chronically HCV-infected patients with high viral titers, in an attempt to infect human hepatocytes. Some stringent conditions of the culture system were required for achieving a successful HCV infection of human hepatocytes (see Methods).

**Figure 1:**
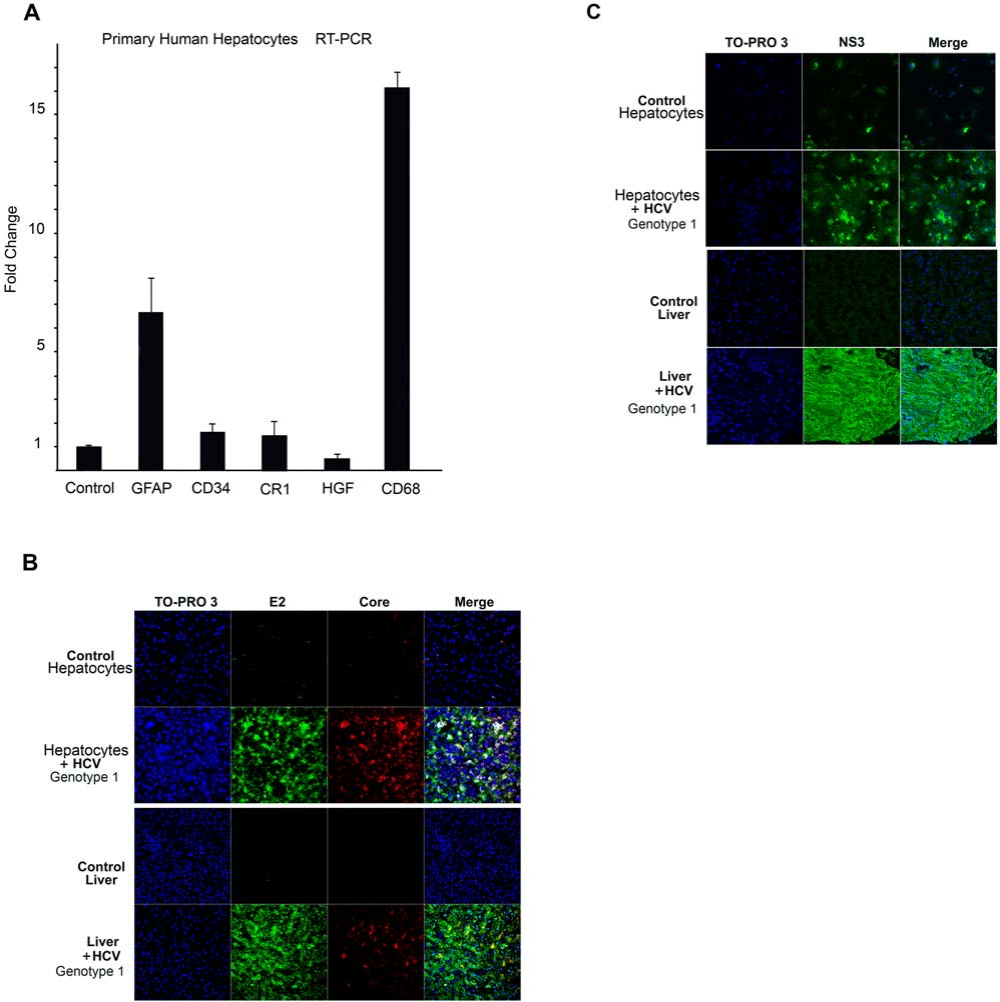
HCV Infection Genotype 1 of the Human Hepatocyte Culture System. A. Day-5 human hepatocyte cultures were assessed by RT-PCR for GFAP, CD-34, CR-1, HGF and CD-68. Values were normalized to control human liver and expressed as fold changes from baseline. GFAP and CD-68 were increased compared to control human liver (*P* <0.05). B. Day-5, primary human hepatocytes infected with HCV genotype 1 (inoculum: 11,200 HCV virions) and control cells, as well as liver from HCV-infected patients or control liver were processed as described in Materials and methods. Scanning confocal laser microscopy was performed for nucleic acids (TO-PRO-3), HCV E-2 and HCV core. Twenty-four hour infected hepatocytes expressed HCV E-2 and core proteins. Control hepatocytes had only background fluorescence for HCV E-2 and core proteins (upper panels). HCV-infected liver expressed also HCV E-2 and core proteins. Control liver hepatocytes had only background fluorescence for HCV E-2 and core proteins (lower panels). Co-localization of HCV E-2 (red) and HCV core (green) is shown in yellow (merge), while co-localization of nucleic acids (blue), HCV E-2 (red) and HCV core (green) is shown in white (merge) in both HCV-infected human hepatocyte cultures and liver . The control hepatocytes had the same amount of control human serum. C. HCV NS3 was determined by scanning confocal laser microscopy in the samples described in (B). Twenty-four hour infected hepatocytes expressed HCV NS3 protein. Control hepatocytes had only background fluorescence for HCV NS3 protein. Co-localization of HCV NS3 (green) is shown in yellow (merge) in HCV-infected human hepatocyte cultures (upper panels) and HCV-infected human liver (lower panels). The control hepatocytes had the same amount of control human serum. Representative results from quadruplicate samples of ten independent experiments with human hepatocytes cultures and seven human liver samples (4 patients with chronic hepatitis C viral infection and severe liver fibrosis and 3 control livers) are shown.

Using these conditions, we attained a robust infection of human hepatocyte cultures with naturally occurring HCV obtained from 33 of 36 consecutive HCV-infected patients, with 3 of 36 failures. HCV infection was achieved in human hepatocyte cultures from 29 different liver donors. Cells were cultured at a high density on a three-dimensional specific collagen type 1 matrix, and in a defined medium without serum, and with liver sinusoidal cells, conditions that allowed hepatocytes to become highly differentiated, recapitulating the physiology of hepatocytes within the liver as we reported previously [14], [18], [28]. On day-5, the human hepatocyte culture system was composed of approximately 95% hepatocytes, and 5% liver sinusoidal endothelial cells and hepatic stellate cells, mimicking the hepatocyte organoid rodent cell culture [29]. As determined by RT-PCR , these uninfected human hepatocyte cultures expressed glial fibrillary acidic protein (GFAP), CD-34, complement receptor-1 (CR1), hepatocyte growth factor (HGF) and CD-68, genes also expressed in a normal liver by hepatic stellate cells, endothelial cells, smooth muscle cells and macrophages [19], [30]–[33]. The RNA values were compared to those of a normal uninfected human liver **(** **Figure 1A** **)**. This may be relevant since these stellate cells and sinusoidal endothelial cells produce HGF, a critical factor for hepatocyte differentiation and survival [31], [34]–[35]. In turn, HGF activates the CCAAT/enhancer binding protein- β (C/EBPβ) [36] which upon phosphorylation induces hepatocyte survival [28].

Naturally occurring HCV genotype 1 infection of human hepatocyte cultures developed rapidly, as reflected by the intense expression of HCV E-2, core and NS3 proteins in the cell layers, after a 24-hr exposure, on laser scanning confocal microscopy using specific antibodies [37] **(** **Figure 1B and 1C** **)**. The HCV E-2 and core proteins were co-localized in the perinuclear region of the human hepatocytes infected with HCV genotype 1, while uninfected control hepatocytes had only background fluorescence **(** **Figure 1B** **)**. This perinuclear localization resembles the previously reported HCV virions in the liver of HCV-infected patients and chimpanzees [38]–[39]. In the HCV-infected hepatocytes, the TO-PRO3 stain for nucleic acids, detected hepatocyte nuclear DNA but also nucleic acids in the cytoplasm of hepatocytes in a ‘salt and pepper’ pattern **(** **Figure 1B** **).** Because this nucleic acid is co-localized with HCV proteins, such as E-2 and core, and it is not expressed either in uninfected control human hepatocyte cultures **(** **Figure 1B** **)**, or in the same hepatocytes cultured in the presence of control human serum, it is not spurious patients' RNA or DNA contaminating the cells or the inoculum, and most likely represents HCV RNA. Moreover, the scanning confocal laser microscopy technique eliminates the possibility of detecting nucleic acids attached to the hepatocyte cell membrane. Further the expression of HCV E-2 and core as well as the cytoplasm nucleic acid was also observed in the liver of HCV-infected patients, but not in control livers **(** **Figure 1B** **).** Another HCV protein, NS3, was also detected in the human hepatocyte cultures infected with HCV genotype 1 after a 24-hr exposure, but not in uninfected control human hepatocyte cultures **(** **Figure 1C** **)**. The expression of HCV NS3 was also observed in the liver of HCV-infected patients, but not in control livers **(** **Figure 1C** **)**. A similar expression of HCV E-2, core and NS3 proteins was also observed in human hepatocyte cultures after a 24-hr infection with HCV genotypes 2 or 3 **(Figure S1)**.

### HCV Amplification in the Human Hepatocyte Culture System

**Figure 2:**
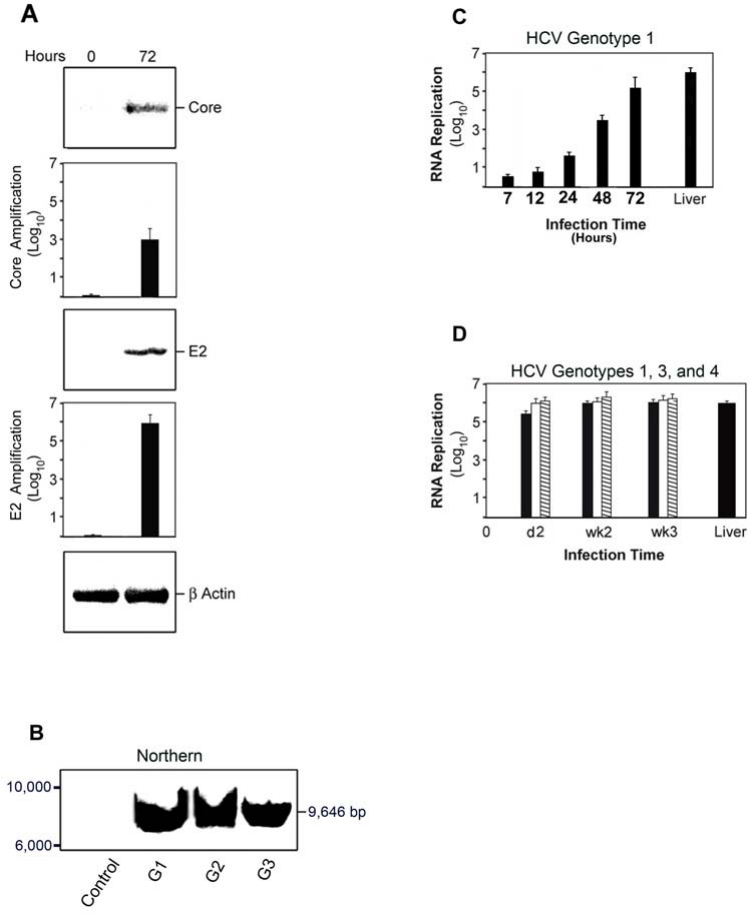
HCV RNA Amplification in the Human Hepatocyte Culture System. Day-5 primary human hepatocytes were infected with HCV genotype 1 (56,000 HCV virions); genotype 2 (68,000 HCV virions); genotype 3 (22,400 HCV virions); or genotype 4 (41,800 HCV virions) for up to week-3 as described in Materials and methods. A. HCV genotype 1 virions in the media were purified by affinity chromatography. Immunoblotting for HCV E-2 and core proteins was done in HCV lysates at time zero (inoculum) and at 72 hr. Immunoblots were quantified on a Kodak 4000 Imaging Station and software as described in Materials and methods. Samples are core at zero and 72 hr ; E-2 at zero and at 72 hr ( *P*<0.05 for E-2 and core) . B. HCV RNA was determined by Northern blot in primary human hepatocytes control or infected with HCV genotype 1 ; genotype 2 and genotype 3 as described in (A). HCV RNA of the expected size is expressed in HCV-infected human hepatocyte cultures, but not in control cells. Results from duplicate samples of three independent experiments are shown. C. HCV RNA was quantified by RT-QPCR in triplicate samples from primary human hepatocytes infected with HCV genotype 1 at 7, 12, 24, 48, and 72 hr, as well as from the livers from two HCV-infected patients (HCV RNA ∼6 log_10_). The HCV RNA increased to ∼3 log_10_ at 48 hr and to ∼5 log_10_ at 72 hr (*P*<0.01). D. HCV RNA was quantified by RT-QPCR in primary human hepatocytes infected with HCV genotype 1 (closed bars); genotype 3 (open bars) and genotype 4 (hatched bars) at day-2, week-2 and week-3, and from livers of two HCV-infected patients (HCV RNA ∼5 log_10_). The HCV RNA increased to ∼5 log_10_ at day-2 and remained at that level at week-2 and week-3 (*P* <0.001). Results from quadruplicate samples of six independent experiments are shown.

Under the conditions that we described, human hepatocyte cultures remained infected for at least 3 weeks. The amplification of HCV genotype 1 infection was analyzed by immunopurifying HCV virions from the medium through HCV E-2 affinity chromatography. The HCV amplification was robust judging by the increased HCV core and E-2 in the medium from time zero (inoculum) to 72 hr **(** **Figure 2A** **)**. Control samples from uninfected hepatocytes lacked detectable HCV core or E-2 proteins.

We evaluated the presence of full-length HCV RNA in the human hepatocyte culture by Northern blot, using specific probes cloned from each donor patient's HCV RNA. The large viral load required for this cloning was obtained from large phlebotomies needed for the treatment of iron overload in patients with Genetic Hemochromatosis that also had HCV infection genotypes 1, 2 and 3. The HCV RNA was of the expected size, and it was detected only in HCV-infected human hepatocyte cultures, but not in uninfected human hepatocyte cultures **(** **Figure 2B** **)**. We also assessed the infection-replication cascade by determining HCV viral particles in the hepatocyte cultures from time zero to 72 hr and from zero to week-3. As detected by quantitative RT-PCR, using modified standard clinical COBAS amplification primers, the HCV RNA increased exponentially up to 5 log_10_ , 72 hr after a HCV genotype 1 infection (*P*<0.01) **(** **Figure 2C** **)** , and remained at a steady-state between 5 log_10_ and 6 log_10_ for up to week-3 for HCV genotypes 1, 3 and 4 (*P*<0.001) **(** **Figure 2D** **)**. Using the same assay protocol, the HCV RNA, corrected by total RNA, was comparable in human hepatocyte cultures after day-2 and in the liver of HCV-infected patients **(** **Figure 2C and 2D** **)**. These data further support the validity of the human hepatocyte culture system to study HCV infection. Although the half-life of HCV virions is less than 5 hours in HCV-infected patients [**40**], it remains to be determined whether the half–life of HCV virions in the hepatocyte culture is similar in the absence of a fully competent immune system.

**Figure 3:**
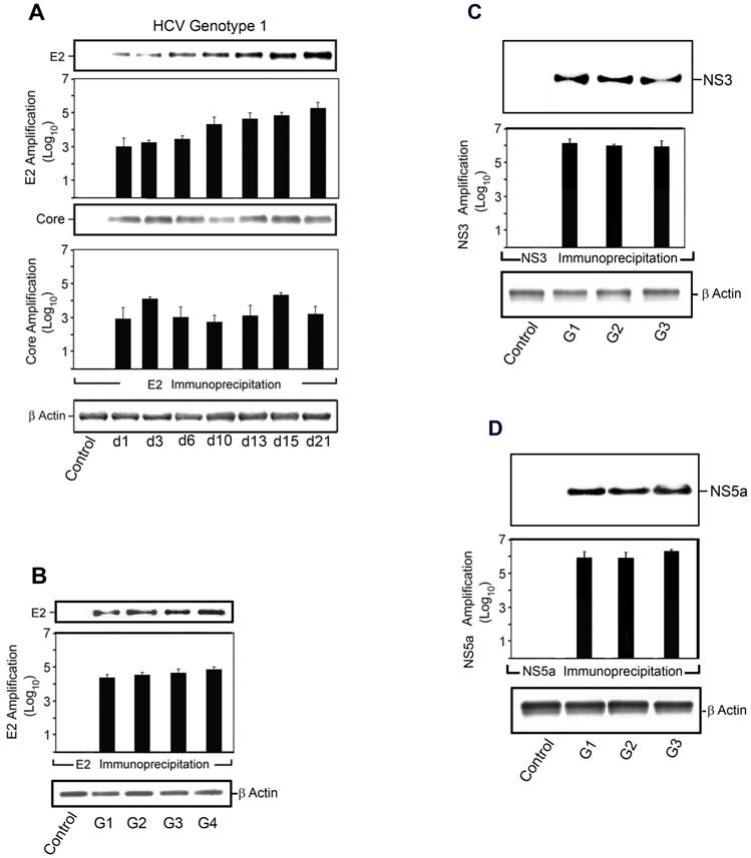
HCV E-2, Core and NS3 Amplification in the Human Hepatocyte Culture System. Day-5 primary human hepatocytes were infected with HCV genotype 1 (56,000 HCV virions); genotype 2 (68,000 HCV virions); genotype 3 (22,400 HCV virions); or genotype 4 (41,800 HCV virions) for up to week-3 as described in Materials and methods. A. HCV E-2 and core were detected by immunoblotting from human hepatocytes cell layers. E-2 and core were expressed in HCV genotype 1-infected hepatocyte cultures for day 1 , day 3 , day 6 , day 10 , day 13 , day 15 , and day 21, compared to control time-zero HCV infection . β-Actin was used as a control for immunoprecipitation. E-2 and core immunoblots shown were quantified on a Kodak 4000 Imaging Station and software; P <0.05 for expression starting on 24 hr when compared to control samples. B. HCV E-2 was detected by immunoblotting from HCV-infected human hepatocytes cell layers. E-2 was expressed in human hepatocytes infected with HCV genotypes 1, 2, 3 and 4 for 24 hr, compared to control at zero time. β-Actin was used as a control for immunoprecipitation. E-2 immunoblots were quantified on a Kodak 4000 Imaging Station and software. The HCV E-2 increased to ∼4 log_10_ at 24 hr for HCV genotypes 1, 2, 3 and 4 (P <0.01). Representative results from triplicate samples of five independent experiments are shown. C. HCV NS3 and NS5a were detected by immunoblotting from HCV-infected human hepatocytes cell layers. NS3 and NS5a were expressed in human hepatocytes infected with HCV genotypes 1, 2, and 3 at day-10, compared to control at zero time. β-Actin was used as a control for immunoprecipitation. NS3 and NS5a immunoblots were quantified on a Kodak 4000 Imaging Station and software. The HCV NS3 and NS5a increased to ∼4 log_10_ for HCV genotypes 1, 2, and 3 (P <0.01). Representative results from triplicate samples of three independent experiments are shown.

Moreover, a similar amplification was detected for HCV proteins E-2, and core up to 21 days in human hepatocytes infected with HCV genotype 1 (*P*<0.05) **(** **Figure 3A** **)**, obtained from a patient chronically infected with HCV. Also, we detected a rapid amplification of HCV genotypes 1, 2, 3 and 4, twenty-four hours after HCV infection judging by E-2 immunopurification (**Figure 3B** **)**. Uninfected, control human hepatocyte cultures did not express either HCV E-2 or core proteins **(** **Figure 3A and 3B** **)**. In addition, expression of HCV NS3 and NS5a was also amplified in human hepatocytes infected with HCV genotypes 1, 2 and 3 **(** **Figure 3C and 3D** **)**. Collectively, these data indicate a robust HCV infection of the normal human hepatocyte culture system for up to 3 weeks **(** **Figures 2** **and** **3** **)**.

The HCV infection of the normal human hepatocyte system was consistent. Sera from 33 of 36 HCV-infected patients successfully infected the normal human hepatocyte culture system. Instability of the HCV in the sera might have negatively affected infection of the human hepatocytes in 3 cases. None of the sera from the uninfected control subjects induced any false positive parameter of HCV infection in the normal human hepatocyte system. The subject population included individuals with chronic HCV infection, viral load >700,000 IU/ml and genotypes 1 (*n*: 21), 2 (*n*: 5), 3 (*n*: 6), or 4 (*n*: 4), but negative for Hepatitis A and B, and HIV. Control sera were obtained from 3 subjects negative for Hepatitis A, B and C, and HIV.

### HCV-Infection of Normal Human Hepatocytes is Dependent on CD-81 and HCV E-2

**Figure 4:**
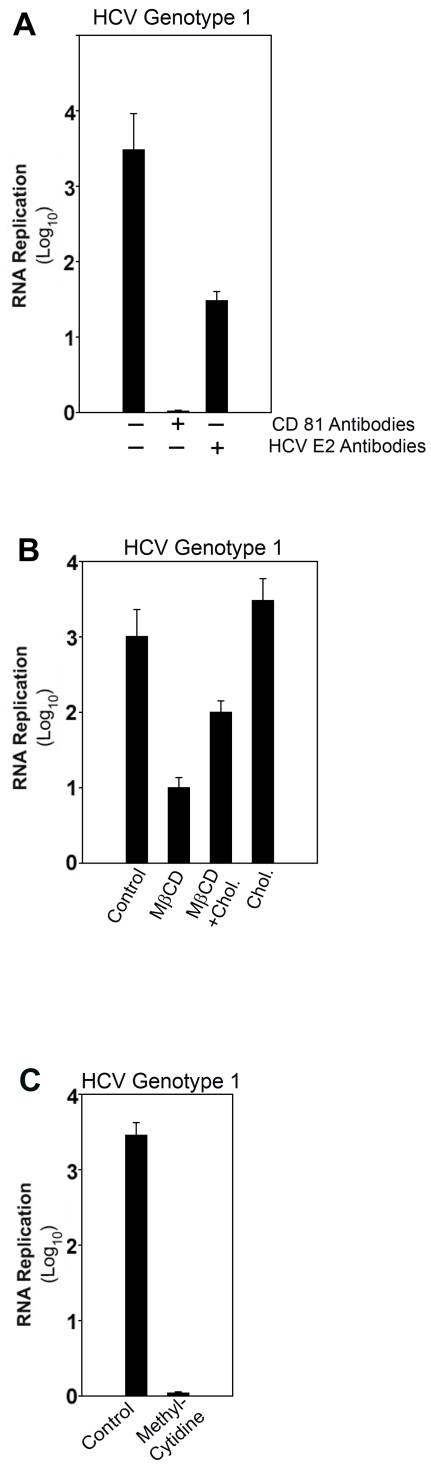
HCV-Infection with Genotype 1 of Normal Human Hepatocytes is Dependent on HCV E-2 and CD-81. Day-5 primary human hepatocytes were infected with HCV genotype 1 (56,000 HCV virions) as described in Materials and methods. HCV RNA replication was determined at 72 hr after infection. A. Human hepatocyte cultures were treated prior to HCV infection without or with antibodies specific to CD-81 and HCV E-2 as described in Materials and methods. Antibodies against CD-81 and HCV E-2 decreased HCV RNA (*P*<0.05). The control hepatocytes had the same amount of control human IgG. B. Human hepatocyte cultures were treated prior to HCV infection without or with MβCD, MβCD and cholesterol, or cholesterol control as described in Materials and methods. Cholesterol depletion with MβCD decreased HCV RNA (*P*<0.05), which was partially rescued by the addition of cholesterol (NS). Treatment with cholesterol alone had no effect on HCV RNA (NS). C. Human hepatocyte cultures were treated prior to and during HCV infection without or with methyl cytidine as described in Materials and methods. Treatment with methyl cytidine decreased HCV RNA (*P<*0.05). Results from triplicate samples are shown.

The mechanisms of hepatitis C viral entry have been extensively investigated. It is known that E-2 dimerizes with E-1, and associates with the cellular CD-81 and the SR-BI receptors, which has proven to be critical for entry of HCV virions [41], [42]. It has also been documented that HCV entry mechanisms involve cholesterol content of the plasma membrane [42]–[45]. Therefore, we assessed whether these mechanisms also are required for HCV infection in normal human hepatocyte cultures. We found that HCV infection of the human hepatocyte culture system with genotype 1 can be blocked with antibodies specific to CD-81 or HCV E-2 **(** **Figure 4A** **)**, or by cholesterol depletion with MβCD **(** **Figure 4B** **)** (*P*<0.05 for all treatments), as reported previously for Huh-7 cells [7] [42], [46]. Further, the HCV E-2 antibodies also inhibited HCV infection with genotypes 2, 3 and 4 (*P*<0.05 for all treatments). Control human IgG did not affect HCV infection **(Figure S2)**.

As expected, the effects of MβCD were rescued by reconstituting cholesterol **(** **Figure 4B** **)**, as reported previously for Huh-7 cells [42]. In addition, HCV replication was inhibited by methyl cytidine (*P*<0.05) **(** **Figure 4C** **)**, as reported previously for Huh-7 cells [47]. The effects of these interventions were determined by measuring HCV RNA. The significant effects of these blockers and inhibitors on HCV infection suggest that the human hepatocyte culture system is physiologically relevant to the study of HCV infection, as previously documented for the Huh-7 cell system.

### The HCV Infection Modulates Interferon-Related Genes in the Human Hepatocyte Culture System

**Figure 5:**
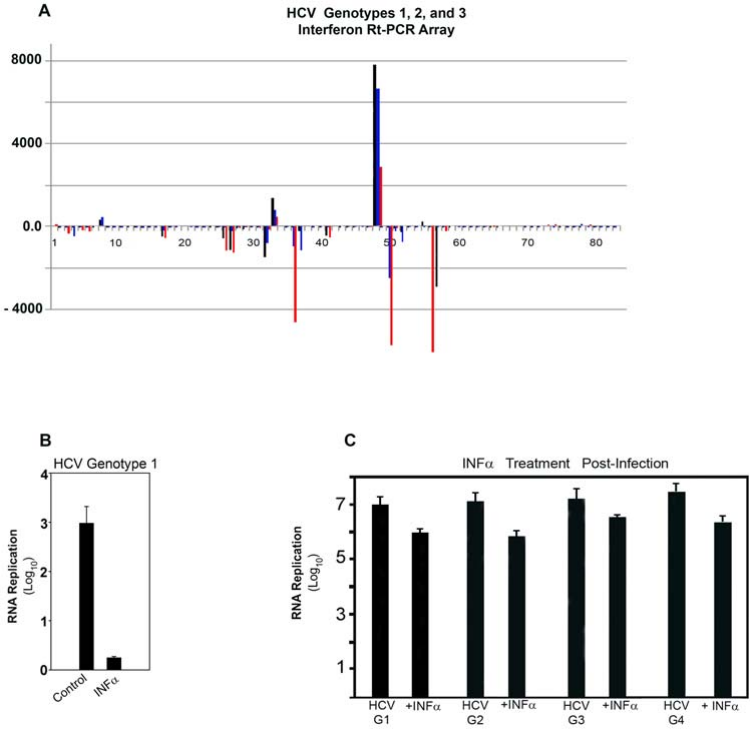
The HCV Infection Modulates Interferon-Related Genes in the Human Hepatocyte Culture System. A. Day-5 primary human hepatocytes were infected with HCV genotype 1 (28,900 HCV virions), genotype 2 (36,200 HCV virions ), or genotype 3 ( 30,800 HCV virions ) as described in Materials and methods. A panel of 83 interferon-related genes was evaluated by RT-QPCR; 22 of these genes were significantly altered (P<0.05 for all genes) compared to control uninfected human hepatocyte cultures . Values for genotypes 1, 2 and 3 are depicted in black, blue and red, respectively. B. Day-5 human hepatocyte cultures were treated 18 hr prior to and for 72 hr during the HCV infection without or with IFN-α as described in Materials and methods. Treatment with IFN-α decreased HCV RNA (*P<*0.05) . C. Day-5 human hepatocyte cultures were treated for 72 hr, 24 hr after the HCV infection with genotypes 1, 2, 3 or 4 without or with IFN-α as described in Materials and methods. Treatment with IFN-α decreased HCV RNA (*P<*0.05 for all genotypes).

Because IFN induction and IFN signaling are important responses to HCV infection [48], we investigated these pathways in the hepatocyte culture system before and after HCV infection. Using a microarray assay to assess expression of 83 interferon-related genes in the human hepatocyte culture system, 72-hr after infection with HCV genotypes 1, 2 or 3, we found a substantially altered gene expression, when compared to uninfected, control human hepatocytes **(** **Figure 5A** **and Table S1)**. Consistent increases were observed after a 72-hr HCV infection for the following genes : i] IL-15 ( # 48); ii] IFN-R 1 (# 33) ; iii] IRF-3 (# 69); iv] IRF-7 ( # 73) ; v] IRF-8 ( # 74) ; vi] IRF-2 binding protein 1 ( # 67) ; vii] IRF-2 binding protein 2 ( # 68); viii] IRF-2 (# 66) ; ix] Adenosine deaminase (# 1) ; and x] Oligoadenylate synthase (# 79) (**Table S1 )**. In contrast, consistent decreases were observed after a 72-hr HCV infection for the following genes : i] IFN-induced Protein 44-like ( # 17); ii] IFN-α 2 (# 27) ; iii] IRF-α 4 (# 29); iv] IRF-α 14 ( # 26) ; v] IRF-α 8 ( # 32) ; vi] IRF-α 6 ( # 31); vii] IRF-α 1 ( # 25); viii] IRF-related Development Regulator 1 (# 41) ; ix] IL-21R (# 50) ; x] IL-22R α ( # 51); xi] IL-28A ( # 52); and xii] Chemokine ligand 10 ( # 6) (**Table S1 )**.

Treatment of human hepatocytes with interferon 18hr before HCV infection, inhibited HCV amplification as measured by RT-PCR for HCV RNA 72 hr later (*P*<0.05) **(** **Figure 5B** **)**. Treatment of human hepatocytes with interferon for 72 hr , 24-hr after the HCV infection with genotypes 1, 2, 3 or 4, inhibited HCV amplification by ∼1 log_10_ (from ∼7 log_10_ ), when compared to untreated HCV-infected human hepatocytes, as measured by RT-PCR for HCV RNA (*P*<0.05) **(** **Figure 5C** **)**. These results further emphasize the physiological relevance of the HCV-infected hepatocyte culture system.

### The Normal Human Hepatocyte Culture System Produces Infectious HCV

**Figure 6:**
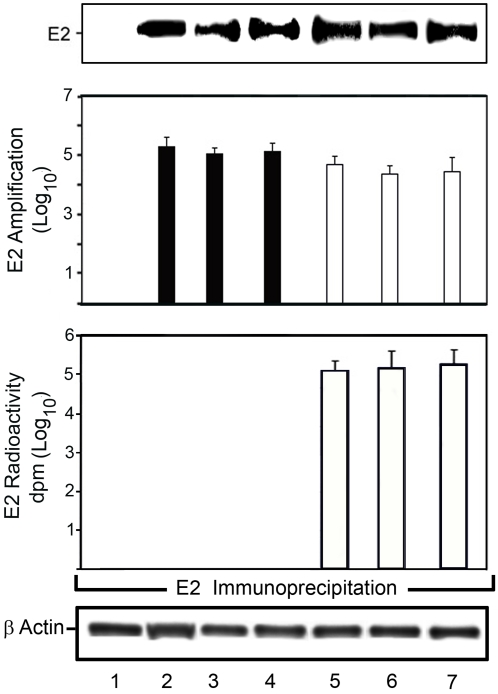
The Human Hepatocyte Culture System Produces Infectious HCV. Day-5 primary human hepatocytes (1.8 million cells/60 cm^2^) were infected with HCV genotype 1 (56,000 HCV virions); genotype 2 (68,000 HCV virions); or genotype 3 (22,400 HCV virions) for 72 hr as described in Materials and methods. Upper panel. HCV E-2 was detected by immunopurification and immunoblotting from cell layers of naïve primary human hepatocytes infected with HCV genotypes 1, 2 or 3 for 72-hr (lanes 5–7) produced by human hepatocytes infected with HCV genotypes 1, 2 or 3 for 72-hr (lanes 2–4) as described in Materials and methods. Control uninfected hepatocytes are shown in lane 1. Middle panel. E-2 immunoblots were quantified on a Kodak 4000 Imaging Station and software; *P* <0.05 for E-2 expression for primary (lanes 2–4) and secondary (lanes 5–7) infections when compared to control samples (lane 1). Results from triplicate samples of two independent experiments are shown. Lower panel. [^35^S]-methionine labeling of HCV E-2 in the secondary infection of human hepatocytes infected with HCV genotypes 1, 2 or 3 (lanes 5–7) produced by the human hepatocyte primary infection. HCV infection was quantified by determining the radioactivity of immunopurified HCV E-2 (*P*<0.01); background radioactivity was negligible. β-Actin was used as a control for immunoprecipitation. Results from triplicate samples of three independent experiments are shown.

HCV virions produced by the primary HCV-infected human hepatocyte cultures were infectious to naïve normal human hepatocyte cultures. The infectivity of naturally occurring HCV virions from HCV-infected patients (into the ‘primary’ culture) and HCV virions produced by normal human hepatocyte cultures (into the ‘secondary’ culture) was comparable **(** **Figure 6** **).** This was judged by the viral amplification as determined by immunopurification of HCV E-2, and quantified either on a Kodak 4000 Imaging Station and software as described [31] (*P<*0.05) **(** **Figure 6** **, upper panel)**, or by the incorporation of [^35^S]-methionine into the newly synthesized HCV E-2 protein in the secondary infection (*P*<0.01) **(** **Figure 6** **, lower panel)**.

Because it has been suggested that infectious HCV virions and infectious Huh-7 produced particles represent a subset banding at a density slightly lower than that of most virions and particles [49]–[51], we isolated HCV virions by isopycnic ultracentrifugation through an iodixanol gradient as described by Yi and coworkers [51]. As described in **Table 1** for HCV genotype 1, the density of the naturally occurring HCV in the serum of the patient fluctuated between 0.979 g/cm^3^ and 1.258 g/cm^3^. Similarly, the densities of the HCV virions in primary and secondary human hepatocyte infection fluctuated between 0.987 g/cm^3^ and 1.298 g/cm^3^, and 0.990 g/cm^3^ and 1.259 g/cm^3^, respectively **(** **Table 1** **)**. The highly infectious HCV virions and particles are believed to concentrate in those with densities below 1.09 g/cm^3^ [50] [51]. In our analysis for HCV genotype 1, approximately 82% of the patient's HCV, 86% of the HCV in the primary infection, and 90% of the HCV in the secondary infection corresponded to those densities, suggesting that they were infectious **(** **Table 1** **)**. The higher HCV densities may correspond to other forms of HCV virions. For example, the nucleocapsid of HCV, isolated by different detergent treatments, was estimated to have a buoyant density of 1.25 g/ml in sucrose [49] , and some clinical HCV samples have densities >1.13 g/ml [49]–[50]

**Table 1:** The Normal Human Hepatocyte Culture System Produces HCV Genotype 1 with a Density of Infectious Virions.

HCV Genotype 1 (Serum)		HCV Genotype 1 (Primary Infection)		HCV Genotype 1 (Secondary Infection)	
Density (g/cm^3^)	RNA (IDV)	Density (g/cm^3^)	RNA (IDV)	Density (g/cm^3^)	RNA (IDV)
**0.979**	8851833	**0.987**	12132120	**0.990**	3451200
**1.031**	9698598	**1.012**	30425472	**1.003**	12611840
**1.049**	6776352	**1.034**	3345300	**1.015**	7651520
**1.067**	5189958	**1.052**	27534276	**1.076**	12516160
**1.258**	6541992	**1.298**	16558080	**1.259**	7376960

HCV genotype 1 virions were isolated by isopycnic ultracentrifugation through an iodixanol gradient as described in Material and methods from patients' serum , from the human hepatocyte primary infection and from the human hepatocyte secondary infection. The densities below 1.09 g/cm^3^ consistent with infectious virions, comprised approximately 82% of the patient's serum, 86% of the primary infection, and 90% of the secondary infection. HCV virions in each fraction were assessed by RT-PCR for HCV RNA , as described in Material and methods.

A similar pattern was observed for HCV genotypes 2 and 3. The percentage of HCV virions that concentrate in densities below 1.09 g/cm^3^ , were approximately 82% and 80% for HCV genotype 2, and 85% and 87% for HCV genotype 3 , in the primary infection and in the secondary human hepatocyte culture system , respectively **(Table S2)**

To our knowledge, this is the first report of a physiologically significant amplification of HCV infection (up to 7 log_10_ ) with naturally occurring genotypes 1, 2 , 3, and 4 in a normal human hepatocyte culture system, suggesting that research with this physiological system may complement that available with the current replicon systems [7]–[9].

## Discussion

The differentiated normal human hepatocyte cell culture described here is a system suitable for investigations of the HCV life cycle, of naturally occurring genotypes 1, 2, 3 and 4 obtained from HCV-infected patients. The infection of normal human hepatocytes was robust for at least 3 weeks and consistent, since normal human hepatocytes from 29 different liver donors were infected with sera from at least one of 33 HCV-infected patients. The HCV amplification achieved in these experiments was up to 7 log_10_ and comparable to that detected in HCV-infected human livers.

HCV infection was validated by seven different approaches: i) time-dependent amplification of newly synthesized HCV RNA (as detected by Northern blot and RT-QPCR ); ii) time-dependent amplification of HCV proteins in cell layer and media (as detected by immunopurification); iii) production of HCV virions that were infectious to naïve human hepatocyte cultures ( as detected by RT-PCR for HCV RNA; HCV E-2 de novo synthesis with [^35^S]-methionine; and E-2 amplification by immunopurification); iv) production of HCV virions with densities below 1.09 g/cm^3^, consistent with highly infectious virions [50]–[51]; v] blockade of HCV infection with antibodies specific to HCV E-2 and CD-81, or cholesterol depletion with MβCD [40]–[42]; vi) IFN-α and methyl cytidine , inhibitors of HCV replication [7], [47] prevented HCV amplification; and vii] induction of a physiological response of interferon-related genes to the HCV infection.

Valuable studies by Fournier, Molina and their coworkers have allowed the culture of HCV in primary human hepatocytes [25]–[27]. However, this culture hepatocyte system had limited efficiency since less than 15% of the sera were infectious [25], the amplification was about 1 log_10_ [25]–[26], the infection declined after 8 days , it was detectable only in the cell layers until day-14, and there was no evidence of the production of infectious virions [25]–[27] . The main apparent differences between the system reported by Fournier, Molina and co-workers [25]–[27], and our system are the following: i] our cellular viability was higher (95% vs. 70–90%) and more stringent (apoptosis and ALT assessment); ii] our cell attachment was induced with higher concentration of fetal calf serum ( 20% vs. 5%) for longer (up to 18 hr vs. 4 hr); iii] in our system, the matrix was rat-tail collagen; iv ] in our system, the collagen matrix was freshly prepared within 24hr of hepatocyte plating, at a concentration of 50 µg/ml or greater; v] in our system, the culture plates were coated with polylysine; and vi] our density plating was higher (1.8 million vs. 0.14 million/60 cm^2^ plate) [25] .

The current Huh-7-derived HCV virions system is simple, but allows replication of only synthetic RNA expressed from selected cloned genomic or subgenomic HCV. Although naïve Huh-7-derived cells can be infected with virions produced by Huh-7 cells transfected with HCV RNA from specific clones, this system cannot be infected with naturally occurring HCV, indicating another major departure of the Huh-7 system from the biology of human HCV infection. In contrast, the normal human hepatocyte system is permissible to infection with naturally occurring HCV genotypes from most patients tested to date, reproducing the high susceptibility of humans to HCV infection of all genotypes [4].

In addition blockers of cell entry and inhibitors of HCV replication [7], [41], [42], [47] prevented HCV replication in the human hepatocyte culture system. Collectively, these results suggest that the normal human hepatocyte culture system mimics some relevant aspects of the infection of hepatocytes by HCV in patients. Therefore, the normal human hepatocyte system may allow high throughput testing of patients' HCV susceptibility to novel drugs, and identification of putative molecular mechanisms within hepatocytes that may explain patients' genetic or acquired resistance to a treatment. For example, hepatocytes cell cultures and HCV could be from donors with various ethnic and genetic backgrounds such as those refractory to HCV treatment (e.g., African Americans). In addition, this hepatocyte system may facilitate early identification of unanticipated cellular targets of novel HCV inhibitors in the context of an HCV infection, possibly, preventing drug-induced liver injury in clinical trials [52]. Further, clinically relevant conditions for HCV infection, such as insulin resistance, fatty liver and iron overload [53]–[54] can be mimicked in these cultures (MB, unpublished observations). In contrast, these physiologically important culture conditions are unlikely to be mimicked in the Huh-7 hepatocellular carcinoma system.

In addition, the human hepatocyte culture system had a physiological response to the HCV infection since many of the 83 interferon-related genes were substantially affected, as reported in the livers of HCV-infected patients [55]. These findings suggest that the human hepatocyte system for HCV infection may contribute to the understanding of the relationship between HCV viral proteins and cellular anti-viral mechanisms, including IFN induction and IFN signaling [48].

The Huh-7 system is capable of generating infectious HCV virions; however, the repertoire is limited with only several HCV chimeric clones available at present [7]. Both the Huh-7 and human hepatocyte systems have comparable high-level replication for a few weeks. While only ∼2% of the replicon Huh-7-derived cells were NS5-positive on day-5, reaching 100% infection on day-24 [8], about 95% of the human hepatocytes were infected with naturally occurring HCV at 24 hr, as indicated by the expression of HCV E-2 and core by confocal scanning laser microscopy. Thus, the human hepatocyte culture system is infected with naturally occurring HCV, at least as rapidly as the Huh-7 cells, mimicking the kinetics of HCV infection in humans. In support of our human hepatocyte HCV infection system, primary hepatocytes from the tree shrew *Tupaia belangeri,* specie susceptible to infection by hepatitis viruses, were also susceptible to HCV infection with sera from HCV-infected patients [56].

Current research uses readily available Huh7-derived hepatocellular carcinoma cells, while the HPV-18/E6E7-human hepatocyte system uses immortalized cells. However, unlike the normal human hepatocytes described in this study, the Huh-7- derived cells are de-differentiated and characterized by abnormal proliferation, deregulated gene expression, dysfunctional mitochondria and aberrant endocytosis and signaling pathways [15]–[18], [20]–[24]. Similarly, the HPV-18/E6E7 immortalized human hepatocytes have an ectopic expression of hTERT [13], which results in aberrant proliferation and could be tumorigenic [24]. It is unknown whether the HPV-18/E6E7 immortalized human hepatocytes are producing infectious HCV virions.

The HCV infection in patients induces proliferation of HCV-infected hepatocytes as determined in liver biopsies by nuclear staining of PCNA (M Buck, unpublished observations). In contrast, the HCV infection system occurs in Huh-7 cancer cells with a high baseline proliferation rate, and does not affect the degree of cell proliferation (M Buck, unpublished observations). Therefore, the differentiated, HCV-infected normal human hepatocyte culture system may help identifying the effects of HCV viral proteins on hepatocyte proliferation and carcinogenesis, signaling pathways, endocytosis, gene expression, and mitochondrial function [14].

The HCV infection of the hepatocyte culture system with naturally occurring HCV virions mimics the biology and kinetics of HCV infection in humans, and produces infectious virions that can infect naïve human hepatocytes. We also determined that most of the HCV virions for genotypes 1, 2, and 3 produced by the primary and secondary human hepatocyte cultures, have a density consistent with those of infectious HCV virions, as proposed previously by Hijikata, Yi and their coworkers [50]–[51]. It remains to be determined by future investigations whether these HCV virions are infectious to chimpanzees and to mice containing human liver grafts as it has been documented with the Huh-7 generated virions [9], [12].

### Clinical Implications

This study suggests that the human hepatocyte culture system described here will complement the Huh-7 virions system in understanding the HCV life cycle, it effects on the hepatocyte, the natural host cell, as well as the possible development of novel therapeutics and vaccines. The human hepatocyte culture system may facilitate studies of the role of insulin resistance and fatty liver on HCV infection since these conditions can be mimicked in the human hepatocytes. Similarly, the mechanisms by which African Americans are refractory to HCV treatment could possibly be analyzed by infecting human hepatocytes from different ethnic backgrounds and studying their response to treatments.

## Materials and Methods

The projects involving human subjects were reviewed and approved by the University of California, San Diego's Human Protection Research Committee.

### Human Primary Hepatocyte Cultures

We obtained hepatocytes (from Tissue Transformation Technologies [Edison, NJ], CellzDirect [Durham, NC], Invitrotechnologies [Baltimore, MD], and BD Biosciences [Woburn, MA]) from anonymous organ donors without liver disease that were not suitable for liver transplantation for technical but not medical reasons. These donors were negative for Hepatitis A, B and C, CMV, HIV, HTLV ½, and RPR-STS.

Hepatocytes were isolated from an encapsulated liver sample by a modified two-step perfusion technique introduced by Seglen [57]. Briefly, the dissected lobe was placed into a custom-made perfusion apparatus and two to five hepatic vessels were cannulated with tubing attached to a multi-channel manifold. A liver fragment (150 to 500 g) was perfused initially (recirculation technique) with calcium-free HBSS supplemented with 0.5 mM EGTA for 20 to 30 min and then with 0.05% collagenase [Sigma] dissolved in L-15 medium (with calcium) at 37°C until the tissue was fully digested. The digested liver was removed, immediately cooled with ice-cold L-15 medium and the cell suspension was strained through serial progressively smaller stainless steel sieves, with a final filtration through 100-micron and 60-micron nylon mesh. The filtered cell suspension was aliquoted into 250-ml tubes and centrifuged three times at 40 g for 3 min at 4°C. After the last centrifugation, the cells were re-suspended, in HypoThermosol-FRS [BioLife Solutions, Inc] combined in one tube and placed on ice.

Cells were centrifuged at 700 rpm for 5 min at 4°C, the supernatant was removed and the cells were washed with Hanks Wash Solution (53.6 mM KCl 0.4 g/l; 4.4 mM KH_2_PO 0.06 g/l; 1.37 M NaCl 8 g/l; 3.4 mM Na_2_HPO_4_ 0.048 g/l; 20 µL CaCl_2_ (2M)) three times. Cells were re-suspended in Hepatocyte Plating Media (500 mL DMEM high glucose; 20% FBS) and plated at a concentration of at 0.625×10^6^ cells/mL. We used diluted collagen (type 1, rat tail-BD Cat. #354236) (50 µg/ml in 0.02N acetic acid) for coating coverslips and plates in about 10ml (enough to cover them) at room temperature for one hour. The collagen solution was then removed and rinsed once with PBS. After the cells attached (<18 hrs), the HPM was replaced by Hepatocyte Media (500 mL DMEM high glucose; 30 mg L-methionine; 104 mg L-leucine; 33.72 mg L-ornithine; 200 µL of 5mM stock dexamethasone; 3 mg Insulin)

### Hepatocyte culturing conditions required for infection with naturally occurring HCV

These conditions include the following: 1) the matrix was rat-tail collagen( BD Biosciences); 2) the collagen matrix was prepared within 24 hr of hepatocyte plating , at a concentration of 50 µg/ml or greater; 3) the culture plates were coated with polylysine; 4) the rinsing of the matrix was minimal; 5) the suspended hepatocytes were allowed to attach in 20% fetal calf serum for not more than 18 hr; 6) the hepatocyte-specific media was given for at least 24 hr prior to the HCV infection; 7) the hepatocytes were >85% confluent until the time of infection; 8) hepatocyte cultures with >5% apoptosis by annexin-V assays and/or increases >3-fold in ALT were discarded; 9) hepatocyte media was changed every 72 hr.

### Human HCV-positive Sera

Sera from 36 HCV-infected patients and 3 control subjects were obtained at the VA San Diego Healthcare System Clinical Laboratory. The subject population included individuals with chronic HCV infection, viral load >200,000 IU/ml and genotypes 1 (*n*: 21), 2 (*n*: 5), 3 (*n*: 6), or 4 (*n*: 4), but negative for Hepatitis A and B, CMV and HIV. The HCV viral load was determined by using the COBAS TaqMan HCV real-time PCR assay (Long Beach VA Medical Center, Long Beach, CA). The dynamic range is 25–100,000,000 IU/mL. Control sera (*n*: 3) were obtained from subjects negative for Hepatitis A, B and C, CMV and HIV. In different experiments, the primary inoculums fluctuated between 3,728 and 68,000 HCV viral particles (maximally 1.2 viral particle/25 hepatocytes). The incubation time for the inoculum was 1 hr.

### Dependence of HCV-Infection of Normal Human Hepatocytes on CD-81 and HCV E-2

Day-5 primary human hepatocytes were infected with HCV genotype 1 (56,000 HCV virions) as described above. Human hepatocyte cultures were treated with antibodies specific to HCV E-2 (25 µg/ml) (BioDesign), CD-81 (25 µg/ml) (Santa Cruz Biotech.) and normal human IgG (25 µg/ml) for 18 hr prior to HCV infection), or by cholesterol depletion with MβCD (7.5 mM for 1 h prior to HCV infection) as described previously [42] [46]. The effects of MβCD were rescued by reconstituting cholesterol (150 µg/ml, 1 hr prior to HCV infection). Also, we used Interferon-α (10 IU/ml for 18 hr prior to and during HCV infection) [7], or methyl cytidine (1 µM for 3 hr prior to and during HCV infection) [47] to inhibit HCV replication. All patient samples obtained, both livers and HCV sera were exempted from informed consent by the IRB, as all identifiers had been removed prior to procurement for the study.

### Infection of Naïve Human Hepatocyte Cultures with Human Hepatocyte Culture-derived HCV

The m.o.i. were for genotype 1: 1 viral particle /25 hepatocytes for the primary infection, and 33 viral particles/25 hepatocytes for the secondary infection; for genotype 2: 1.2 viral particle/25 hepatocytes for the primary infection, and 44 viral particles/25 hepatocytes for the secondary infection; and genotype 3: 0.4 viral particle/25 hepatocytes for the primary infection, and 33 viral particles/25 hepatocytes for the secondary infection. Naïve day-5 primary human hepatocytes were cultured with 20 µl of cell layer lysates (estimated to be comparable to the original inoculums or approximately 1 viral particle/25 hepatocytes) from the day 10 post-infection primary HCV-infected human hepatocyte cultures that had 3 media changes prior to harvest. HCV RNA was determined on primary and secondary infections on day-10 and day-7, respectively, after Equilibrium Ultracentrifugation of HCV particles (see below), as described above. In other experiments naïve human hepatocytes were cultured in a methionine-free medium for 24 hr. After this period, hepatocytes were infected for 72-hr as above, but in the presence of 100 µCi [^35^S]-methionine (>1,000Ci/mMol) (MP Biomedicals). HCV E-2 was immunopurified from cell layers, immunoblotted and the E-2 bands were excised and counted using a Beckman LS 6500 liquid scintillation counter.

### Immunoprecipitation and Immunoblotting

HCV E-2, HCV core, and β-actin were detected by immunoblotting the immunoprecipitates from hepatocyte lysates as described [58] following the chemiluminescence protocol (DuPont) and using purified IgG antibodies as described [59]. Immunoblots were quantified on a Kodak 4000 Imaging Station and software as described [31].

### HCV RNA Determination

Total RNA was isolated from HCV infected primary human hepatocytes using the TRizol Reagent from Invitrogen (California) following the manufacturers protocol. DNA digestion was performed with TURBO DNase from Ambion (Texas) following the manufacturers protocol. The cDNA synthesis was performed using AffinityScript RT and Fermentas Rnase Inhibitor from Stratgene (California), per manufacturers protocol., using HCVDNAprimer (*5′-ATGACCTTACCCAAATTGCG-3′) or a random 9-mer (5′-NNNNNNNNN-3′) using 0.5–2.0 mg RNA with 2 pMol gene specific primer or random 9-mer.* PCR was performed using Qiagen's (California) HotStar Taq DNA Polymerase. Primers were: NCR5: 5′-CCTCCCGGGAGAGCCATAGT and NCR3: 5′-ACAAGGCCTTTCGCGAACCCAA. The amplicon was ∼150 bp. PCR conditions were: Cycle Temp time 1 cycle 95° 15 min, 58–60 cycles; 94° 20 sec, 55° 30 sec, 72° 30 sec,1cycle 10° ∞. Gel Analysis was performed on a 2% TBE gel run at 100v for 15–20 min. An Apex 100 bp-Low DNA Ladder from Genesee Scientific (California) was used. Results were compared to HCV infected liver samples run in tandem.

### Confocal Microscopy

Fluorescent labels were observed using a triple-channel fluorescence microscope or a confocal microscope. Fluorochromes utilized included TOPRO-3 (blue), Alexa 488 (green) and Alexa 594 (red) (Molecular Probes). The percentage of HCV infected hepatocytes was determined by confocal microscopy using HCV E-2 (BioDesign), Core(BioDesign), and NS3 (Santa Cruz Biotech.) specific antibodies [19] [60]. At least 100 cells were analyzed per experimental point [28]. We analyzed the nuclear morphology by staining cells with TOPRO-3 (R&D Systems). Two observers analyzed each immunofluorescent study independently, with an intra-observer agreement of >90%.

### Affinity Column Chromatography

Catch and Release affinity columns and protocol (Upstate) were used with HCV E-2 (Biodesign), NS3 (Santa Cruz Biotech.), and NS5a (Santa Cruz Biotech.) specific antibodies with non-denaturing buffers as specified by the manufacturer. This method was more efficient and specific in purifying HCV virions than the standard immunoprecipitation techniques. Negative and positive control samples were run in parallel.

### Equilibrium Ultracentrifugation of HCV particles

The density of HCV in the human hepatocyte culture system was performed as described by Yi et al [51]. Primary infections were incubated for 10 days and Secondary infections for 7 days. Cells were harvested, supernatants were collected and clarified by low-speed centrifugation, and concentrated ∼10-fold using a Centricon PBHK Centrifugal Plus-20 filter unit with an Ultracel PL membrane (100-kDa exclusion) (Millipore, Billerica, MA) and then layered on top of a preformed, continuous 10 to 40% iodixanol (OptiPrep, Sigma-Aldrich, St. Louis, MO) gradient prepared in Hanks balanced salt solution (Invitrogen, Carlsbad, CA). Gradients were centrifuged in a Beckman SW60 rotor (Beckman Coulter, Fullerton, CA) at 45,000 rpm for 16 h at 4°C, and nine fractions (500 µl each) were collected from the top of the tube. The density of each fraction was estimated by weighing a 100-µl aliquot of each fraction.

### Interferon PCR Array

The human Interferon and receptor Array that was used was from SuperArray (Frederick, MD). RNA Isolation was performed as above using Invitrogen's Trizol Reagent. DNA Digestionwas performed as above using Ambion's TURBO™ DNase. After a rigorous DNA digestion, Chloroform Extraction was performed followed by a Phenol:Chloroforme extraction. cDNA synthesis was achieved using Strategene’s Protocols and reagents as above. Random 9-mer 5′-NNNNNNNNN-3′, Fermentas Rnase Inhibitor and Stratagene's AffinityScript RT, 0.5–2.0 ug RNA, and 270 ng random 9-mer were used. 20 uL cDNA reaction mixtures with 91 uL nuclease-free water was used in the RT2 Profiler™ PCR Array System following the manufacturer's protocol. The array was run on a Bio-Rad iQ5 Multicolor Real-Time PCR Detection System and data analysis was performed using the excel spreadsheet provided by SuperArray.

### Northern Blot

In order to generate specific probes for a Northern of our infected culture systems, it was necessary to clone the sequences from the serum of each of the HCV viral donors. There would be no probes available that had sufficient specificity for our individual donors due to the known genetic drift of the HCV virus. Virus isolation was performed using BioVintage ( California) Opti-Q-1 Virus Isolation kit. RNA Isolation was done using Invitrogen Trizol Reagent. For cDNA Synthesis we used: HCVcDNAprimer 5′-ATGACCTTACCCAAATTGCG, Fermentas Rnase Inhibitor ,AffinityScript RT, with 0.5–2.0 ug RNA, 2 pmol gene specific primer, 40 U RNase Inhibitor, and 100 mM DTT at 42°C for 60 min. For the PCR, we used Qiagen's HotStarTaq DNA Polymerase, 10 uL of diluted (25-fold) cDNA reaction mixture: (500 uL H20 added to the 20 uL cDNA reaction mixture). To generate the **HCVg1 probe** we used HCVsensePrimer5 5′- gCgACACTCCACCATgAATC and HCVantisensePrimer5 5′- TTCCgCAgACCACTATg. The amplicon was 139 bp. The conditions were; 1cycle 95° 15 min, 60cycles 95° 30 sec, 55° 30 sec, 68° 30 sec, 1cycle 10° ∞. To generate the **HCVg3** and **HCVg4 probes** we used (RT-PCR antisense#2) 5′- CACTCgCAAgCACCCTATCA and (RT-PCR sense) 5′- TTCAgCCAgAAAgCgTCTAg. The expected amplicon was 259 bp. The conditions used were: 1cycle 95° 15 min, 60cycles 95° 30 sec, 55° 30 sec, 68° 30 sec, and 1cycle 10° ∞. PCR purification was performed using Qiagen's QIAquick PCR Purification Kit. The amplicon was ligated to Promega's pGEM T-easy vector at a ratio of Vector : Insert 1 : 2 using a Roche Rapid Ligation kit. The ligation was transformed into Promega's JM109 cells andplated onto LB+Carbenicillin+X-gal+IPTG plates. Plasmids were isolated using Qiagen's QIAprep Spin Miniprep Kit. Resulting plasmids were sequenced by the CFAR Molecular Biology Core (http://molbiocore.ucsd.edu/). The templates were linearized with Pst I (NEB), and SpeI (NEB)[ **sense** strand synthesis], and Not I (NEB), and Sph I (NEB)[ **antisense** strand synthesis]. Digests were analyzed on a 1% TAE gel. Linearized plasmids were treated with Proteinase K (100–200 ug/mL Proteinase K and 0.5% SDS for 30 min at 50°C), and phenol:chloroform extracted. Probes were generated from the linearized plasmids using Ambion's MAXIscript Kit, 500 uM total (for each ATP, GTP, UTP), 3.125 uM total for CTP, 1 ug DNA template, and Cytidine 5′-Triphosphate, ([a-32P], 6000 Ci/mmol, MP BioMedicals). 1 uL Ambion's TURBO DNase was used to digets the template DNA. Removal of unincorporated nucleotides was achieved with NucAway Spin Columns. The Northern Blot was prepared with Ambion's NorthernMax –Gly. 30 ug of Total RNA was loaded into gel with Sigma Aldrich RNA ladder. Gel was run at 5V/cmand transferred using a Schleicher & Schuell Turboblotter onto Schleicher & Schuell Nytran N membrane and Crosslinked with Fisher Brand UV Crosslinker. Hybridization was performed with 1×10^6^ cpm/mL probe using Ambion's NorthernMax –Gly protocol. The hybridized membrane was exposed to BlueDevil Film from Genesee Scientific. Sigma's Transcript RNA Markers (0.28–10 kb) were used.

### RT-PCR for GFAP, CD34, CR1, HGF, and CD68

RNA Isolation was performed using Invitrogen Trizol Reagent as above. Ambion's TURBO™ DNase was used for DNA digestion. Following rigorous DNA digestion and phenol:chloroform extraction, cDNA synthesis was performed using Strategene's Protocol and Random 9-mer 5′-NNNNNNNNN-3′, Fermentas Rnase Inhibitor, and Stratagene's AffinityScript RT with 2.0 ug RNA, and 270 ng random 9-mer. The qRT-PCR Master Mix was SuperArray's RT^2^ qPCR Master Mixes and the primers were:RT^2^ qPCR Primer Assay for Human GFAP: PPH02408E, RT^2^ qPCR Primer Assay for Human CD34: PPH02455E, RT^2^ qPCR Primer Assay for Human CR1: PPH17118A, RT^2^ qPCR Primer Assay for Human HGF: PPH00163B and RT^2^ qPCR Primer Assay for Human CD68: PPH05574E. The protocol was from SuperArray; **RT2 qPCR Primer Assays,** Real-Time RT-PCR Gene Expression Analysis, Part #1016A Version 1.5 6/22/2007, pgs. 13-14.

### Human Livers

We obtained anonymous, de-identified liver samples from 4 patients with chronic hepatitis C viral infection and severe liver fibrosis (Metavir scores of 3 or 4) (53+/−17 years) and from 3 control subjects without liver disease (60+/−13 years) (NDRI).

### Statistical Analysis

Results are expressed as mean (±SD). Either the Student-*t* or the Wilcoxon Mann-Whitney tests were used to evaluate the differences of the means between groups for parametric and non-parametric populations, respectively, with a *P* value of <0.05 as significant.

## Supporting Information

Figure S1 HCV Infection Genotypes 2 and 3 of the Human Hepatocyte Culture System. A. Day-5, primary human hepatocytes infected with HCV genotypes 2 or 3 (inoculums: 28,700 and 44,300 HCV virions, respectively) and control cells were processed as described in Materials and methods. Scanning confocal laser microscopy was performed for nucleic acids (TO-PRO-3), HCV E-2, HCV core and HCV NS-3 as described in Fig 1. Twenty-four hours infected hepatocytes expressed HCV E-2, core and NS-3 proteins. Control hepatocytes had only background fluorescence for HCV E-2 and core proteins (upper panels). Co-localization of HCV E-2 (red) and HCV core (green) is shown in yellow (merge), while co-localization of nucleic acids (blue), HCV E-2 (red) and HCV core (green) is shown in white (merge) in HCV-infected human hepatocyte cultures. Co-localization of HCV NS3 (green) is shown in yellow (merge) in HCV-infected human hepatocyte cultures (lower panels). Control hepatocytes received control human sera. Representative results from triplicate samples of three independent experiments with human hepatocytes cultures are shown.
(1.32 MB TIF)

Figure S2 HCV-Infection with Genotypes 2, 3 and 4 of Normal Human Hepatocytes is Dependent on HCV E-2. Day-5 primary human hepatocytes were infected with HCV genotype 2 (42,600 HCV virions), genotype 3 (37,800 HCV virions) and genotype 4 (62,500 HCV virions ) , as described in Materials and methods. HCV RNA replication was determined at 72 hr after infection. Human hepatocyte cultures were treated prior to HCV infection without or with antibodies specific to HCV E-2 as described in Materials and methods. Antibodies against HCV E-2 decreased HCV RNA for all genotypes (P<0.05). The control hepatocytes had the same amount of control human IgG.
(0.25 MB TIF)

Table S1 Quantitative Changes in Interferon-Related Genes in the Human Hepatocyte Culture System Infected with HCV Genotype 1. Day-5 primary human hepatocytes were infected with HCV genotype 1 (38,100 HCV virions), as described in Materials and methods. A panel of 83 interferon-related genes was evaluated by RT-QPCR; 22 of these genes were significantly altered (P<0.05 for all genes) compared to control uninfected human hepatocyte cultures . Ten genes were increased ( # 1; # 33; # 48; # 66; # 67; # 68; # 69 ; # 73; # 74; and # 79) , while twelve genes were decreased ( # 6; # 17; # 25; # 26; # 27; # 29; # 31; # 32; # 41; # 50; # 51; and # 52).
(0.12 MB DOC)

Table S2 The Human Hepatocyte Culture System Produces HCV Genotypes 2 and 3 with a Density of Infectious Virions. HCV genotypes 2 and 3 virions were isolated by isopycnic ultracentrifugation through an iodixanol gradient as described in Material and methods from the human hepatocyte primary infection and from the human hepatocyte secondary infection. The densities below 1.09 g/cm3 consistent with infectious virions, comprised approximately 82% and 80% for genotype 2, and 85% and 87% for genotype 3, of the primary infection or the secondary infection , respectively .
(0.03 MB DOC)
