# Supplementary figures and images for: Direct Infection and Replication of Naturally Occurring Hepatitis C Virus Genotypes 1, 2, 3 and 4 in Normal Human Hepatocyte Cultures
Source: PLoS One. 2008 Jul 16;3(7):e2660. doi: 10.1371/journal.pone.0002660 (PMC2442186; doi:10.1371/journal.pone.0002660)

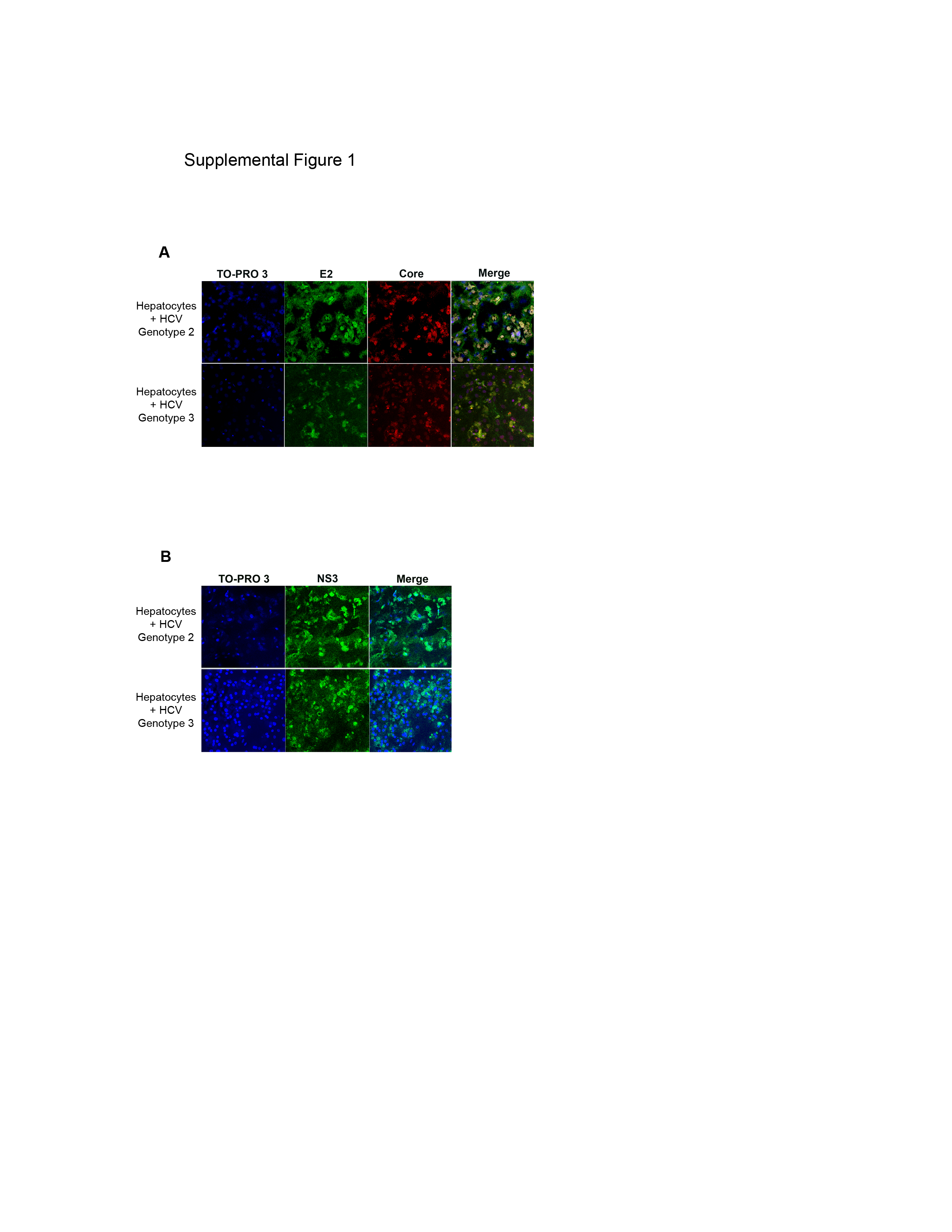

Supplement: Figure S1 — HCV Infection Genotypes 2 and 3 of the Human Hepatocyte Culture System. A. Day-5, primary human hepatocytes infected with HCV genotypes 2 or 3 (inoculums: 28,700 and 44,300 HCV virions, respectively) and control cells were processed as described in Materials and methods. Scanning confocal laser microscopy was performed for nucleic acids (TO-PRO-3), HCV E-2, HCV core and HCV NS-3 as described in Fig 1. Twenty-four hours infected hepatocytes expressed HCV E-2, core and NS-3 proteins. Control hepatocytes had only background fluorescence for HCV E-2 and core proteins (upper panels). Co-localization of HCV E-2 (red) and HCV core (green) is shown in yellow (merge), while co-localization of nucleic acids (blue), HCV E-2 (red) and HCV core (green) is shown in white (merge) in HCV-infected human hepatocyte cultures. Co-localization of HCV NS3 (green) is shown in yellow (merge) in HCV-infected human hepatocyte cultures (lower panels). Control hepatocytes received control human sera. Representative results from triplicate samples of three independent experiments with human hepatocytes cultures are shown. (1.32 MB TIF) [file pone.0002660.s001.tif]

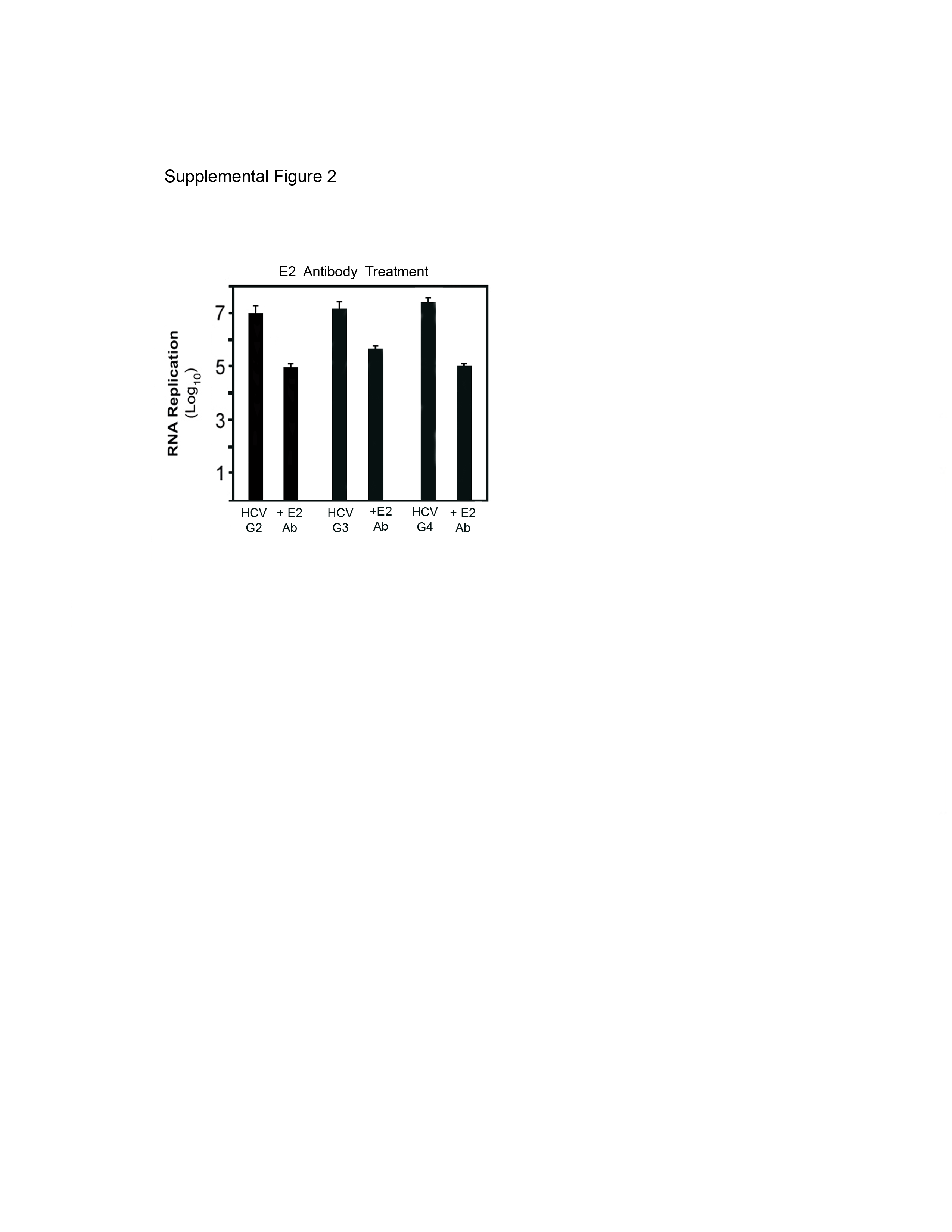

Supplement: Figure S2 — HCV-Infection with Genotypes 2, 3 and 4 of Normal Human Hepatocytes is Dependent on HCV E-2. Day-5 primary human hepatocytes were infected with HCV genotype 2 (42,600 HCV virions), genotype 3 (37,800 HCV virions) and genotype 4 (62,500 HCV virions ) , as described in Materials and methods. HCV RNA replication was determined at 72 hr after infection. Human hepatocyte cultures were treated prior to HCV infection without or with antibodies specific to HCV E-2 as described in Materials and methods. Antibodies against HCV E-2 decreased HCV RNA for all genotypes (P<0.05). The control hepatocytes had the same amount of control human IgG. (0.25 MB TIF) [file pone.0002660.s002.tif]
