# Supplementary material for: Direct Infection and Replication of Naturally Occurring Hepatitis C Virus Genotypes 1, 2, 3 and 4 in Normal Human Hepatocyte Cultures
Source: PLoS One. 2008 Jul 16;3(7):e2660. doi: 10.1371/journal.pone.0002660 (PMC2442186; doi:10.1371/journal.pone.0002660)
Supplement: Table S1 — Quantitative Changes in Interferon-Related Genes in the Human Hepatocyte Culture System Infected with HCV Genotype 1. Day-5 primary human hepatocytes were infected with HCV genotype 1 (38,100 HCV virions), as described in Materials and methods. A panel of 83 interferon-related genes was evaluated by RT-QPCR; 22 of these genes were significantly altered (P<0.05 for all genes) compared to control uninfected human hepatocyte cultures . Ten genes were increased ( # 1; # 33; # 48; # 66; # 67; # 68; # 69 ; # 73; # 74; and # 79) , while twelve genes were decreased ( # 6; # 17; # 25; # 26; # 27; # 29; # 31; # 32; # 41; # 50; # 51; and # 52). (0.12 MB DOC) [file pone.0002660.s003.doc]

| **Number** | **Symbol** | **Name** | **Fold Regulation**  **Genotype 1**  **Genotype 2**  **Genotype 3** |
| --- | --- | --- | --- |
| **1** | ADAR1/DRADA | Adenosine Deaminase, RNA-Specific | 31.56  23.75  22.47 |
| **2** | MGC1774 | Ciliary Neurotrophic Factor Receptor | 1.04  -2.35  -7.31 |
| **3** | CRL2/CRLF2Y | Cytokine Receptor-like Factor 2 | -6.19  -41.93  -388.02 |
| **4** | CD116/CDw116 | Colony Stimulating Factor 2 Receptor, alpha | -1.54  -487.75  -1.64 |
| **5** | CD114/GCSFR | Colony Stimulating Factor 3 Receptor | -3.84  -1.88  -200.85 |
| **6** | C7/IFI10 | Chemokine (C-X-C motif) Ligand 10 | -14.83  -65.80  -266.87 |
| **7** | EBI3 | Epstein-Barr Virus Induced Gene 3 | 7.41  10.27  -9.71 |
| **8** | CD142/TF | Coagulation Factor III | 282.09  430.54  -2.30 |
| **9** | DIRS1/FNDC6 | Interleukin 20 Receptor beta | 4.20  2.59  2.91 |
| **10** | G1P2/IFI15 | ISG15 Ubiquitin-like Modifier | -2.04  -1.91  -2.97 |
| **11** | 6-16/FAM14C | Interferon Alpha-Inducible Protein 6 | -2.99  -6.96  -16.68 |
| **12** | IFNGIP1/PYHIN2 | Interferon Gamma-Inducible Protein 16 | 6.02  7.52  6.59 |
| **13** | FAM14D/ISG12 | Interferon Alpha-Inducible Protein 27 | -3.36  -4.38  -7.31 |
| **14** | GILT/IFI-30 | Interferon Gamma-Inducible Protein 30 | 2.41  2.66  1.75 |
| **15** | IFP35 | Interferon-Induced Protein 35 | 2.51  3.39  1.47 |
| **16** | MTAP44/p44 | Interferon-Induced Protein 44 | -2.14  -55.72  -4.72 |
| **17** | C1orf29/GS3686 | Interferon-Induced Protein 44-like | -498.00  -200.85  -596.34 |
| **18** | HLcd/IDDM19 | Interferon-Induced with Helicase C Domain 1 | 3.51  -3.51  3.84 |
| **19** | G10P1/GARG-16 | Interferon-Induced Protein with Tetratricopeptide Repeats 1 | -7.21  -8.11  -5.54 |
| **20** | DKFZp781M1841 | Interferon-Induced Protein with Tetratricopeptide Repeats 1-like | 12.38  34.30  2.62 |
| **21** | G10P2/GARG-39 | Interferon-Induced Protein with Tetratricopeptide Repeats 2 | 9.65  -1.01  4.63 |
| **22** | CIG-49/GARG-49 | Interferon-Induced Protein with Tetratricopeptide Repeats 3 | 4.17  -29.04  2.50 |
| **23** | 9-27/CD225 | Interferon Induced Transmembrane Protein 1 (9-27) | 1.21  -1.53  -2.11 |
| **24** | 1-8D | Interferon Induced Transmembrane Protein 2 (1-8D) | 7.94  9.58  7.84 |
| **25** | IFL/IFN | Interferon Alpha 1 | -25.63  -4.23  -7.78 |
| **26** | MGC125756 | Interferon Alpha 14 | -592.22  -31.48  -1209.34 |
| **27** | IFNA/INFA2 | Interferon Alpha 2 | -1128.35  -216.77  -1296.13 |
| **28** | MGC126687 | Interferon Alpha 21 | -81.57  1.68  -120.26 |
| **29** | INFA4 | Interferon Alpha 4 | -128.89  -24.93  -104.69 |
| **30** | INFA5 | Interferon Alpha 5 | -6.15  5.86  -11.31 |
| **31** | IFNA 6 | Interferon Alpha 6 | -12.47  -53.45  -125.37 |
| **32** | IFNA8 | Interferon Alpha 8 | -1499.22  -797.86  -174.85 |
| **33** | AVP/IFN-alpha-REC | Interferon (alpha, beta, and omega) Receptor 1 | 1323.37  786.88  404.50 |
| **34** | IFN-R/IFN-alpha-REC | Interferon (alpha, beta, and omega) Receptor 2 | 11.31  8.22  7.11 |
| **35** | IFB/IFF | Interferon Beta 1 | -20.68  -29.86  -11.55 |
| **36** | IFNT1/PRO655 | Interferon Epsilon 1 | -3.25  -982.29  -4640.29 |
| **37** | CD119/IFNGR | Interferon Gamma Receptor 2 | -225.97  -1176.27  -2.95 |
| **38** | AF-1/IFGR2 | Interferon Gamma Receptor 2 | 28.64  6.45  7.36 |
| **39** | RP11-27J8.1 | Interferon Kappa | 4.47  5.74  5.70 |
| **40** | IFNW1 | Interferon Omega 1 | 1.21  3.36  -1.91 |
| **41** | PC4/TIS7 | Interferon-related Development Regulator 1 | -461.44  -58.49  -552.56 |
| **42** | IFNRP/SKMc15 | Interferon-related Development Regulator 2 | 17.63  39.12  12.73 |
| **43** | CDW210A/HIL-10R | Interleukin 10 Receptor alpha | 5.90  9.00  3.16 |
| **44** | CDW2110B/CRF2-4 | Interleukin 10 Receptor beta | -4.44  -4.82  -17.88 |
| **45** | MGC2146 | Interleukin 11 Receptor alpha | 3.32  -1.09  1.32 |
| **46** | CLMF/CLMF2 | Interleukin 12B | 30.48  5.13  15.14 |
| **47** | CD213A1/IL13Ra | Interleukin 13 Receptor alpha | -4.66  -3.89  -93.70 |
| **48** | IL15 | Interleukin 15 | 7804.01  6653.97  2817.11 |
| **49** | IL-20R1/ZCYTOR7 | Interleukin 20 Receptor alpha | -4.00  -3.97  -8.40 |
| **50** | NILR | Interleukin 21 Receptor | -18.90  -2503.97  -5752.61 |
| **51** | CRF2-10/CRF2-S1 | Interleukin 22 Receptor alpha 2 | -98.36  -227.54  -121.94 |
| **52** | INFL2/IL-28A | Interleukin 28A (interferon lambda 2) | -265.03  -781.44  -34.54 |
| **53** | CRF2/12 | Interleukin 28 Receptor alpha (interferon lambda receptor) | -1.58  -1.85  -4.26 |
| **54** | IFNL1/IL-29 | Interleukin 29 (interferon lambda 1) | 3.61  -2.38  1.55 |
| **55** | CD122/P70-75 | Interleukin 2 Receptor beta | 188.71  16.34  -2.30 |
| **56** | CD132/IMD4 | Interleukin 2 Receptor gamma | -10.93  -4.08  -6122.09 |
| **57** | CRL/CRL3 | Interleukin 31 Receptor A | -2936.74  -2.19  -6.11 |
| **58** | CD123/IL3R | Interleukin 3 Receptor alpha | -8.94  -1.65  -233.94 |
| **59** | CD124/IL4RA | Interleukin 4 Receptor | 2.27  -1.72  -6.45 |
| **60** | CD125/CDw125 | Interleukin 5 Receptor alpha | 27.67  41.07  15.35 |
| **61** | BSF2 | Interleukin 6 (interferon beta 2) | -1.14  -2.73  -3.29 |
| **62** | CD126/IL-6R-1 | Interleukin 6 Receptor | -1.89  -2.81  -5.24 |
| **63** | CD127/CDW127 | Interleukin 7 Receptor | -1.13  -1.68  -2.99 |
| **64** | CD129 | Interleukin 9 Receptor | -4.20  -2.97  -6.02 |
| **65** | IRF-1/MAR | Interferon Regulatory Factor 1 | 1.68  -1.56  -42.22 |
| **66** | DKFZp686FO244/IRF-2 | Interferon Regulatory Factor 2 | 6.19  8.34  3.92 |
| **67** | DKFZP434M154 | Interferon Regulatory Factor 2 Binding Protein 1 | 13.45  22.63  9.32 |
| **68** | MGC72189 | Interferon Regulatory Factor 2 Binding Protein 2 | 33.13  39.67  9.99 |
| **69** | IRF-3 | Interferon Regulatory Factor 3 | 17.75  14.72  2.39 |
| **70** | LSIRF/MUM1 | Interferon Regulatory Factor 4 | 6.68  6.54  4.50 |
| **71** | IRF5 | Interferon Regulatory Factor 5 | -3.14  -1.22  -1.62 |
| **72** | LPS/OFC6 | Interferon Regulatory Factor 6 | 1.56  -2.03  1.91 |
| **73** | IRF-7H/IRF7A | Interferon Regulatory Factor 7 | 31.78  5.90  29.04 |
| **74** | H-ICSBP/ICSBP | Interferon Regulatory Factor 8 | 24.76  35.26  27.86 |
| **75** | IFI1/IRGM1 | Immunity-related GTPase Family M | -1.22  1.44  -1.82 |
| **76** | CD295/OBR | Leptin Receptor | 2.83  6.50  3.73 |
| **77** | C-MPL/CD110 | Myeloproliferative Leukemia Virus Oncogene | 4.03  1.96  4.20 |
| **78** | IFI-78K/IFI78 | Myxovirus Resistance 1, Interferon-inducible Protein p78 | 1.39  -1.84  -1.21 |
| **79** | IFI-4/OIAS | 2”,5’-Oligoadenylate Synthetase 1 | 32.00  85.63  38.85 |
| **80** | IFI5111/PA28A | Proteosome Activator Subunit 1 | 2.38  1.49  1.97 |
| **81** | IFIX | Pyrin and HIN Domain Family, member 1 | 3.81  4.53  3.73 |
| **82** | IFI41/IFI175 | SP110 Nuclear Body Protein | -1.92  -1.00  -2.30 |
| **83** | CMD1G/CMH9 | Titin | 2.64  2.66  2.08 |

Supplemental Table 1.
