# Supplementary material for: Direct Infection and Replication of Naturally Occurring Hepatitis C Virus Genotypes 1, 2, 3 and 4 in Normal Human Hepatocyte Cultures
Source: PLoS One. 2008 Jul 16;3(7):e2660. doi: 10.1371/journal.pone.0002660 (PMC2442186; doi:10.1371/journal.pone.0002660)
Supplement: Table S2 — The Human Hepatocyte Culture System Produces HCV Genotypes 2 and 3 with a Density of Infectious Virions. HCV genotypes 2 and 3 virions were isolated by isopycnic ultracentrifugation through an iodixanol gradient as described in Material and methods from the human hepatocyte primary infection and from the human hepatocyte secondary infection. The densities below 1.09 g/cm3 consistent with infectious virions, comprised approximately 82% and 80% for genotype 2, and 85% and 87% for genotype 3, of the primary infection or the secondary infection , respectively . (0.03 MB DOC) [file pone.0002660.s004.doc]

| **HCV Genotype 2 (Primary Infection)** | | **HCV Genotype 2 (Secondary Infection)** | |
| --- | --- | --- | --- |
| **Density (g/cm^3^)** | RNA (IDV) | **Density (g/cm^3^)** | RNA (IDV) |
| **0.975** | 28031745 | **0.981** | 1758400 |
| **1.028** | 39215715 | **1.016** | 10195840 |
| **1.047** | 31566885 | **1.033** | 3125760 |
| **1.058** | 30022755 | **1.054** | 10697920 |
| **1.137** | 28199325 | **1.193** | 6298880 |

| **HCV Genotype 3 (Primary Infection)** | | **HCV Genotype 3 (Secondary Infection)** | |
| --- | --- | --- | --- |
| **Density (g/cm^3^)** | RNA (IDV) | **Density (g/cm^3^)** | RNA (IDV) |
| **0.987** | 9236304 | **0.9773** | 10063872 |
| **1.014** | 30292080 | **1.0250** | 41209344 |
| **1.045** | 30137184 | **1.0190** | 38071296 |
| **1.057** | 29518104 | **1.0373** | 18205344 |
| **1.174** | 18132576 | **1.1533** | 16466976 |

Supplemental Table 2.
